# Inhalation Conscious Sedation with Nitrous Oxide and Oxygen as Alternative to General Anesthesia in Precooperative, Fearful, and Disabled Pediatric Dental Patients: A Large Survey on 688 Working Sessions

**DOI:** 10.1155/2016/7289310

**Published:** 2016-09-26

**Authors:** Angela Galeotti, Annelyse Garret Bernardin, Vincenzo D'Antò, Gianmaria Fabrizio Ferrazzano, Tina Gentile, Valeria Viarani, Giorgio Cassabgi, Tiziana Cantile

**Affiliations:** ^1^Division of Dentistry and Orthodontics, Bambino Gesù Hospital, Viale San Paolo 15, 00146 Rome, Italy; ^2^Department of Neuroscience, Reproductive and Oral Sciences, Section of Paediatric Dentistry, University of Naples, Federico II, Via Pansini 5, 80131 Naples, Italy

## Abstract

*Aim*. To evaluate the effectiveness and the tolerability of the nitrous oxide sedation for dental treatment on a large pediatric sample constituting precooperative, fearful, and disabled patients.* Methods*. 472 noncooperating patients (aged 4 to 17) were treated under conscious sedation. The following data were calculated: average age; gender distribution; success/failure; adverse effects; number of treatments; kind of dental procedure undertaken; number of dental procedures for each working session; number of working sessions for each patient; differences between males and females and between healthy and disabled patients in relation to success; success in relation to age; and level of cooperation using Venham score.* Results*. 688 conscious sedations were carried out. The success was 86.3%. Adverse effects occurred in 2.5%. 1317 dental procedures were performed. In relation to the success, there was a statistically significant difference between healthy and disabled patients. Sex and age were not significant factors for the success. Venham score was higher at the first contact with the dentist than during the treatment.* Conclusions*. Inhalation conscious sedation represented an effective and safe method to obtain cooperation, even in very young patients, and it could reduce the number of pediatric patients referred to hospitals for general anesthesia.

## 1. Introduction

Dental fear, anxiety related to the dental procedures, and hypersensitivity to pain have been recognized to be an obstacle to the successful treatment in children, impeding, or even precluding, quality dental care [1, 2].

Different surveys showed that the prevalence of dental anxiety in children and adolescents ranges from about 5% to about 24% all over the world [3–5].

Furthermore, dental fear, anxiety, and low pain tolerance are associated with increased levels of caries [6]. In fact, fear of the dentist or behavior management problems can result in untreated dental caries [7].

In particular, a recent study, investigating the prevalence of clinical consequences of untreated dental caries and its relation to dental fear, showed that children with high dental fear had 2.05 times the risk of untreated caries as compared to children with low fear [8].

Pain and suffering due to untreated diseases can lead to problems in eating and speaking and attending to learning [9].

Young, fearful, and uncooperative pediatric dental patients should be managed with behavioral techniques [10].

However, although behavioral techniques could be useful in reducing anxiety, there is a part of the pediatric patients that are not able to tolerate dental procedures and may require alternative approaches, such as conscious sedation with nitrous oxide and oxygen or general anesthesia [7].

General anesthesia is the most common modality for managing uncooperative children [11]. However, the morbidity and mortality risks associated with general anesthesia are considerably higher compared with conscious sedation [12]. Furthermore, the discomfort produced and the inconvenience of a prolonged time of no oral feeding make general anesthesia a no longer recommended “best practice” for dental care [13–15].

Moreover, costs for conscious sedation are estimated to be cheaper by about a third compared with general anesthesia [16].

Conscious sedation is a technique in which the use of a drug or drugs produces a state of depression of the central nervous system enabling treatment to be carried out, but during which verbal contact with the patient is maintained throughout the period of sedation. The level of sedation must be such that the patient remains conscious, retains protective reflexes, and is able to understand and to respond to verbal commands [17]. It may be considered as the first level in the sedation process [18, 19].

Nitrous oxide (N_2_O) is a colorless and virtually odorless gas with a faint, sweet smell. It is an effective analgesic/anxiolytic agent causing central nervous system (CNS) depression and euphoria with little effect on the respiratory system [20].

The technique uses subanesthetic concentrations of nitrous oxide delivered with oxygen from dedicated machinery via a nasal mask. Nitrous oxide is poorly soluble with a high minimum alveolar concentration; rapid onset of action is therefore coupled with a rapid recovery period; the duration of the sedation is controlled and the patient can quickly return to normal activities [21].

Moreover, the efficacy of inhalatory sedation with N_2_O has been studied in groups with a low mean age for pediatric medical procedures, but not for dental care. Studied populations for what concerns dental treatment were individuals with disability or large groups of children and adults and not solely children of young age [22, 23].

In the light of these considerations, the aim of this study was to investigate the effectiveness and the tolerability of the nitrous oxide sedation during dental treatment on a large pediatric sample constituting precooperative and fearful patients with low pain tolerance and of disabled patients as an alternative to general anesthesia for providing high quality dental health care.

## 2. Materials and Methods

An observational survey was performed at the Bambino Gesù Pediatric Hospital, Division of Dentistry and Orthodontics, Rome, Italy, from January 2014 to December 2014. The study was conducted in accordance with the Declaration of Helsinki and it was approved by the local Hospital Authority. Verbal and written explanations of the procedures were given to the parents of the patients. A written consent was signed by them.

A large sample of 472 referred pediatric patients (ASA I and II) unable to accept dental procedures (precooperative children, patients with dental phobia and low pain tolerance, and patients with intellectual disability), aged 4 to 17, were treated under conscious sedation (Master Flux Plus, Tecno-Gaz, Italy).

Exclusion criteria were severe obstructive pulmonary disease; severe emotional disturbances or drug-related dependencies; acute otitis media; and recent tympanic drainage.

Patients were asked not to eat for at least 2 hours before conscious sedation treatment.

Heart rate, oxygen saturation, and blood pressure were monitored at the beginning of the dental treatment, every 10 minutes during the procedure, and at the end of the treatment.

Parents were invited to be present in the room throughout the dental procedure performed with the aid of conscious sedation.

At the start, 100% oxygen was delivered via a flavored nasal mask for 1 to 2 minutes and then nitrous oxide, from a concentration of 30%, was titrated in 5–10% increments to the maximum desired level for each patient by appropriately trained and experienced dentists with the aid of dental nurses, until adequate sedation was achieved (patients should be quiet and nearly motionless but able to understand and to respond to verbal commands). A flow rate of 4 to 9 L/min was generally used.

All personnel involved in patient care was required to have current training in basic life support and in advanced cardiac life support.

After an induction period of 8 minutes, dental treatment was carried out according to a predetermined treatment plan, while verbal contact with the patient was maintained.

All pediatric patients were responsive to verbal command throughout the duration of the treatment.

During the procedure, the patients were reminded to breathe through the nose in order for the gas to work. At the end of the treatment, 100% oxygen was administered for 3–5 min.

The patient's physical status and alertness were assessed before discharge using the Aldrete score [24].

Dose and time of administration of local anesthesia and inspired concentration of oxygen and nitrous oxide were also recorded.

The following data were calculated: the average age of the patients treated and the gender distribution; the overall success/failure; the percentage of successful sessions both for healthy and for disabled patients; the percentage of adverse effects occurring; the overall number of treated teeth and the kind of dental procedure undertaken during the study; the number of dental procedures carried out for each working session; the number of working sessions for each patient; and the level of cooperation, using the modified Venham scale.

The Venham scale is a six-point scale, ranging from 0 (that means a relaxed children) to 5 (that indicates a children out of control). These scores were recorded at 5 time intervals: TC: at first contact with the dentist; T0: at the start of the induction; T1: at the end of the induction; T2: during the first injection of local anesthesia; and T3: during dental treatment [25].

Differences between male and female patients and between healthy and disabled patients in relation to success/failure were defined by Chi-square analysis.

Furthermore, the success/failure was analyzed in relation to mean age using a one-way analysis of variance with a standard *F*-test. For binary variables a 95% confidence interval for the success between groups was calculated; for continuous variables a 95% confidence interval for the difference in mean scores between the groups was calculated.

The data were, then, entered into a database and analyzed with Statistical Package for Social Science (SPSS).

## 3. Results

At the end of the survey 472 pediatric patients, aged 4 to 17 (for a total of 688 working sessions), were treated under conscious sedation.

**Figure 1:**
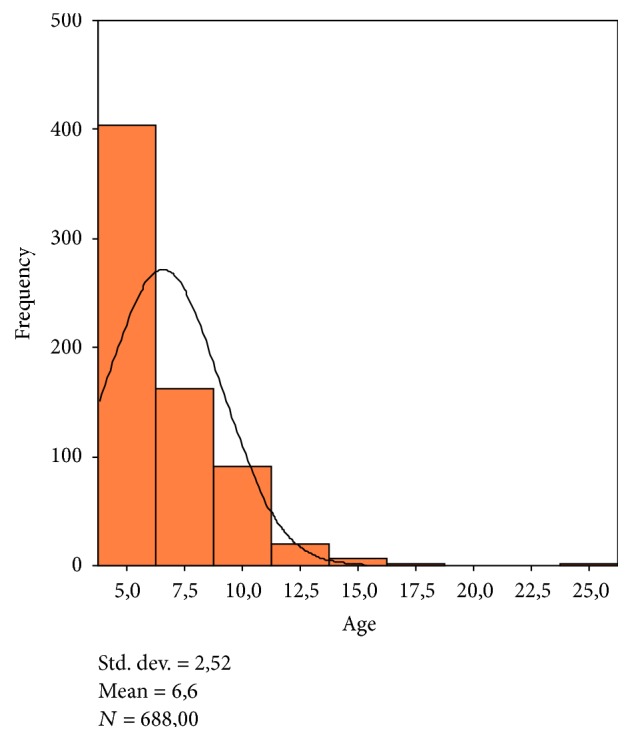
Age distribution.

The mean age was 6.57 ± 2.52. In relation to the age distribution, 58.7% of working sections were performed on patients aged between 4 and 6 and 72.1% on patients younger than 8 years. The age distribution was summarized in Figure 1.

The overall percentage of successful sessions was 86.3%.

The mean age for the dental sessions in which patients successfully completed the treatment was 6.63 ± 2.53; the mean age for the dental sessions in which patients did not successfully complete the treatment was 6.19 ± 2.46. In relation to the mean age, there was no statistically significant difference between success and failure.

In relation to the gender, 336 (48.8%) working sessions were performed on female patients and 352 (51.2%) on males patients.

Of the 336 working sessions performed on female patients, 296 were successfully completed, while in 40 sessions failure occurred.

Of the 352 working sessions performed on male patients, 298 were successfully completed, while in 54 sessions failure occurred.

Chi-square analysis showed that, in relation to the success/failure, there was no statistically significant difference between males and females.

In relation to the disability, 628 (91.3%) working sessions were performed on healthy patients and 60 (8.7%) on patients with intellectual disability.

**Figure 2:**
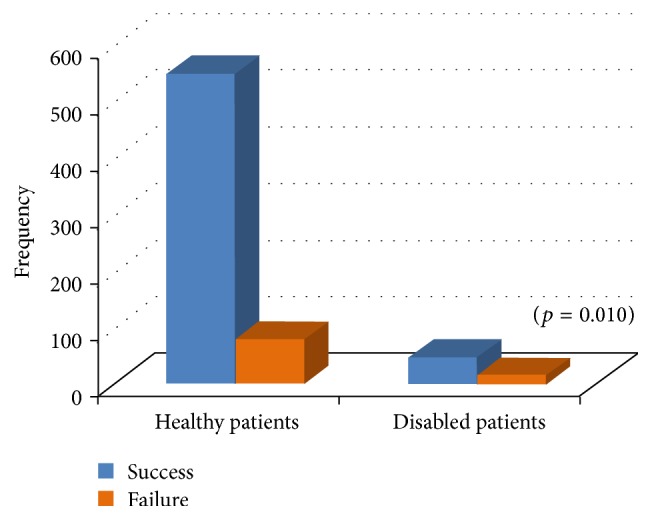
Success/failure in relation to healthy and disabled children.

Of the 628 working sessions performed on healthy patients, 549 were successfully completed, while in 79 sessions failure occurred (Figure 2).

Of the 60 working sessions performed on disabled patients, 45 were successfully completed, while in 15 sessions failure occurred (Figure 2).

Chi-square analysis showed that, in relation to the success/failure, there was a statistically significant difference between healthy and disabled patients (*p* = 0.010).

**Figure 3:**
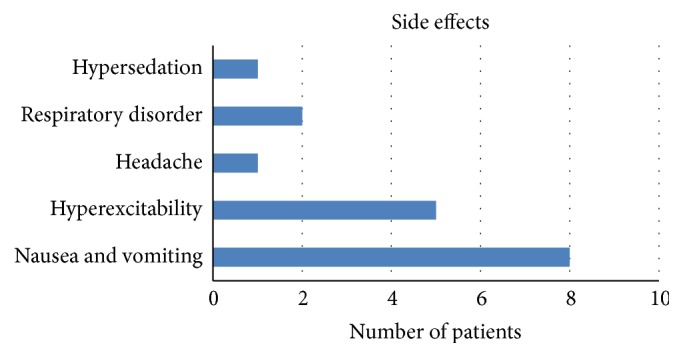
Adverse effects distribution.

Adverse effects occurred in 2.5% of all case, the most frequent symptoms were nausea and vomiting (1.2%). The adverse effects distribution was summarized in Figure 3.

**Figure 4:**
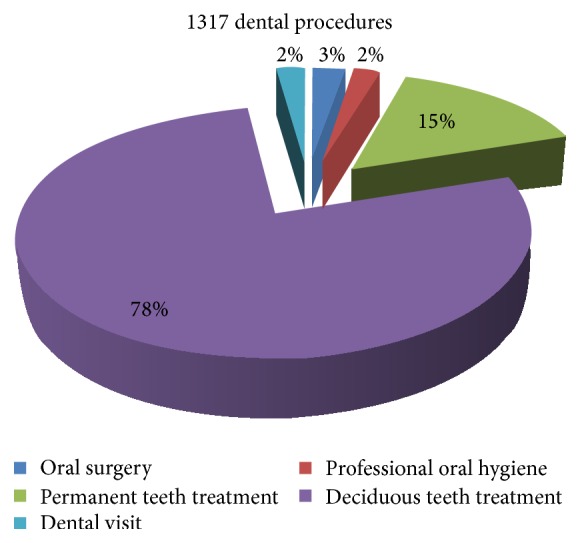
Dental treatments undertaken during the working sessions with oxygen and nitrous oxide sedation.

During the study, 1317 dental procedures were performed: 1024 were treatments on deciduous teeth; 202 were treatments on permanent teeth; 30 were dental visit; 34 were oral surgery; and 27 were professional oral hygiene treatments (Figure 4).

**Figure 5:**
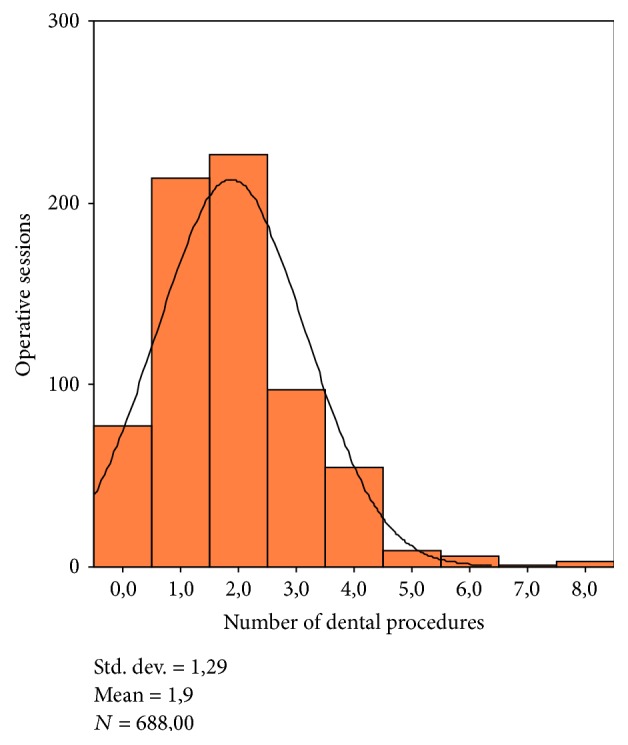
Number of dental procedures for each operative session.

The mean number of dental procedures carried out for each working session was 1.9 ± 1.29. The distribution of these results was summarized in Figure 5.

**Figure 6:**
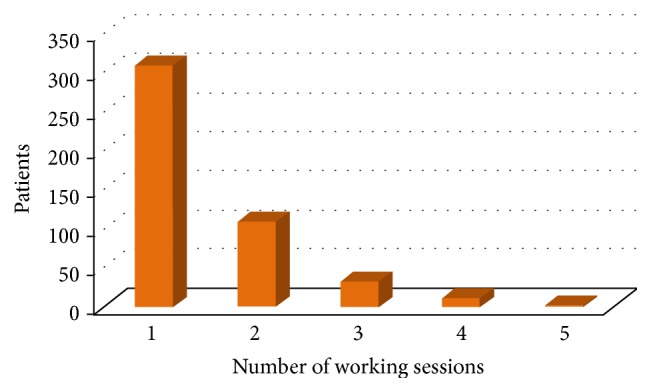
Number of working sessions for each patient.

The mean number of working sessions for each patient was shown in Figure 6.

The modified Venham scale, used to assess the level of cooperation, gave the following results.

**Figure 7:**
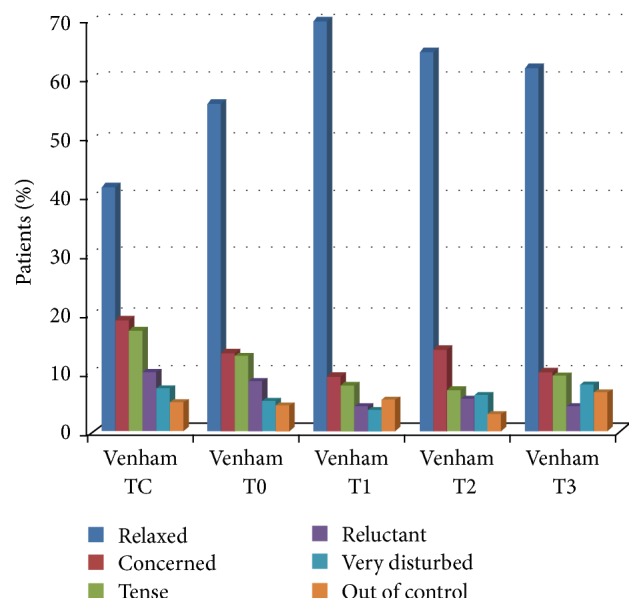
Level of cooperation according to the modified Venham scale.

At first contact with the dentist (TC) the mean score was 1.36 ± 1.51; at the start of the induction (T0) the mean score was 1.06 ± 1.48; at the end of the induction (T1) the mean score was 0.77 ± 1.43; during the first injection of local anesthesia (T2) the mean score was 0.83 ± 1.39; during dental treatment (T3) the mean score was 1.06 ± 1.62. At the first contact with the doctor only about 40% of children were relaxed. This percentage increased at the end of induction and slightly decreased during the injection of local anesthesia and during dental treatment (Figure 7).

## 4. Discussion

The results of this study showed that conscious sedation with nitrous oxide and oxygen can be effectively used for providing high quality dental health care in a large pediatric sample constituting precooperative and fearful patients and of disabled patients, who fail to accept dental treatment, in alternative to general anesthesia.

In this study, the overall percentage of successful sessions was 86.3%. These results are comparable with other studies, reporting success rates of 93% and 83.9%, respectively [15, 26].

Analysis of the results showed that in the present survey the mean age was 6.57 ± 2.52, with 58.7% of working sections performed on patients aged between 4 and 6. Therefore, in this study patients subjected to dental procedures in conscious sedation were younger than patients recruited in other studies [26].

Furthermore, in relation to the mean age, in this study, there was no statistically significant difference between success and failure. These results were in contrast with Foley [26] who reported that, comparing those patients who successfully completed treatment with those for whom treatment was abandoned, the successful cases were older and this was statistically significant. Our data can be interpreted in an encouraging way, having achieved in the present study a considerable success percentage even in precooperative patients.

Regarding the disability, even if of the 60 working sessions performed on disabled patients, 45 were successfully completed, in relation to the success/failure, there was a statistically significant difference between healthy and disabled patients (*p* = 0.010). This could be explained considering that disability, impairing communications, intellectual functioning, and linguistic development made it difficult to provide quality dental care. In fact, a disabled patient cannot be able to breathe adequately through a nasal mask or to tolerate unpleasant and long dental procedures. Therefore, only when patients demonstrated a total lack of cooperation, the use of general anesthesia was justified.

In fact, with conscious sedation being safer than general anesthesia [27], it should be considered the first choice management treatment. In addition, in a review conducted by Lyratzopoulos and Blain, the authors affirmed that morbidity associated with inhalation sedation is minor and infrequent with respect to general anesthesia [16].

Holroyd declared that conscious sedation was a viable and cost-effective alternative to general anesthesia for children requiring extractions, especially orthodontic extractions. Instead, in the present study, dentist performed several types of dental procedures, both in deciduous and in permanent dentition, expanding the field of use, with the aim of restoring all aspects of oral health [21].

During the working session, the percentage of nitrous oxide delivered did not exceed 50% that represented the maximum concentration recommended by the American Academy of Pediatric Dentistry in order to avoid nitrous oxide adverse effects [20]. Furthermore, to guarantee high safety standards, during the procedures patients were monitored with the aid of pulse oximetry, allowing for continuous monitoring of heart rate and blood oxygen saturation.

In relation to adverse effect occurring, the percentage was found to be very low (2.5%). The most frequent symptoms were nausea and vomiting (1.2%), in accordance with other authors who reported nausea in the 1% of all cases [28].

Finally, in relation to the level of cooperation assessed using the modified Venham scale, the mean score during the first injection of local anesthesia was lower than the mean score registered at the first contact with the dentist. This result could be explained with the analgesic/anxiolytic effect of nitrous oxide sedation, allowing reduction or elimination of pain, anxiety, and discomfort, enabling treatment to be carried out satisfactorily.

## 5. Conclusion

Oral health is directly related to general health and wellbeing of pediatric patients, especially those with disabilities and those with behavioral management problems, because they have greater oral health needs. Although it can be a challenge, all pediatric patients should be able to expect painless, high quality dental care, maximizing comfort and cooperation.

The evidence from this large survey suggests that this technique may be a useful alternative to general anesthesia (GA), even in precooperative children, and it could reduce the number of pediatric patients referred to hospitals for GA. The use of conscious sedation with nitrous oxide resulted in successful completion of dental treatment in 86. 3% of cases.

Conscious sedation can be considered safe, practical, and effective both for pediatric very young and fearful patients with low pain tolerance and for patients with intellectual disability.
